# Supplementary figures and images for: Ecological drift during colonization drives within-host and between-host heterogeneity in an animal-associated symbiont
Source: PLoS Biol. 2024 Apr 25;22(4):e3002304. doi: 10.1371/journal.pbio.3002304 (PMC11075893; doi:10.1371/journal.pbio.3002304)

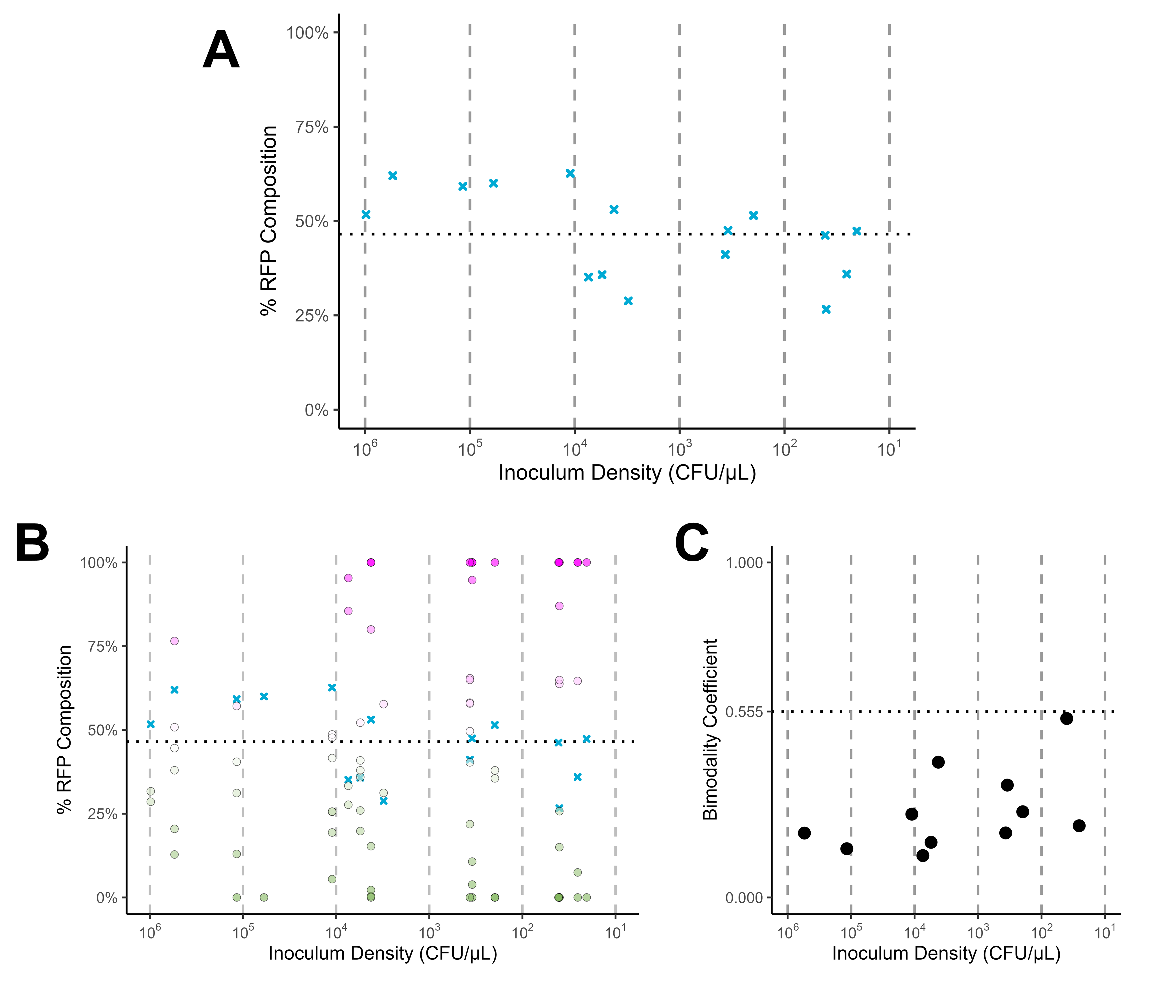

Supplement: S1 Fig — The data underlying this figure can be found in S2 Data. (A) Relative GA-OX1 RFP abundance within inocula used in isogenic colonization trials. Vertical lines demarcate which trials were aggregated for analysis in Fig 1. Blue X marks indicate the inoculum density, and the percent relative abundance of GA-OX1 RFP, in each trial. The dotted horizontal line represents the average relative abundance of GA-OX1 RFP (46.5%) across all trials. (B) Variable colonization outcomes associated with different transmission bottleneck sizes in isogenic co-inoculation, disaggregated from Fig 2B. Blue X marks indicate the inoculum density, and the percent relative abundance of GA-OX1 RFP, in each trial. Points indicate successfully colonized nymphs associated with each inoculation trial, and the color of each point and its position along the y-axis represent the percent relative abundance of GA-OX1 RFP colonies among all fluorescent colonies recovered from each nymph. Magenta points represent insects containing only RFP colonies, green points represent insects containing only GFP colonies, and faded magenta/green colonies are co-colonized. Note that multiple points overlap, particularly at the extremes of 0% and 100% RFP composition, due to the absence of jittering. (C) Bimodality coefficients calculated from unaggregated trials. Bimodality coefficients (black points) calculated from data in panel B. The 0.555 threshold (marked with a dotted line) indicates the bimodality coefficient expected from a uniform distribution. (TIF) [file pbio.3002304.s001.tif]

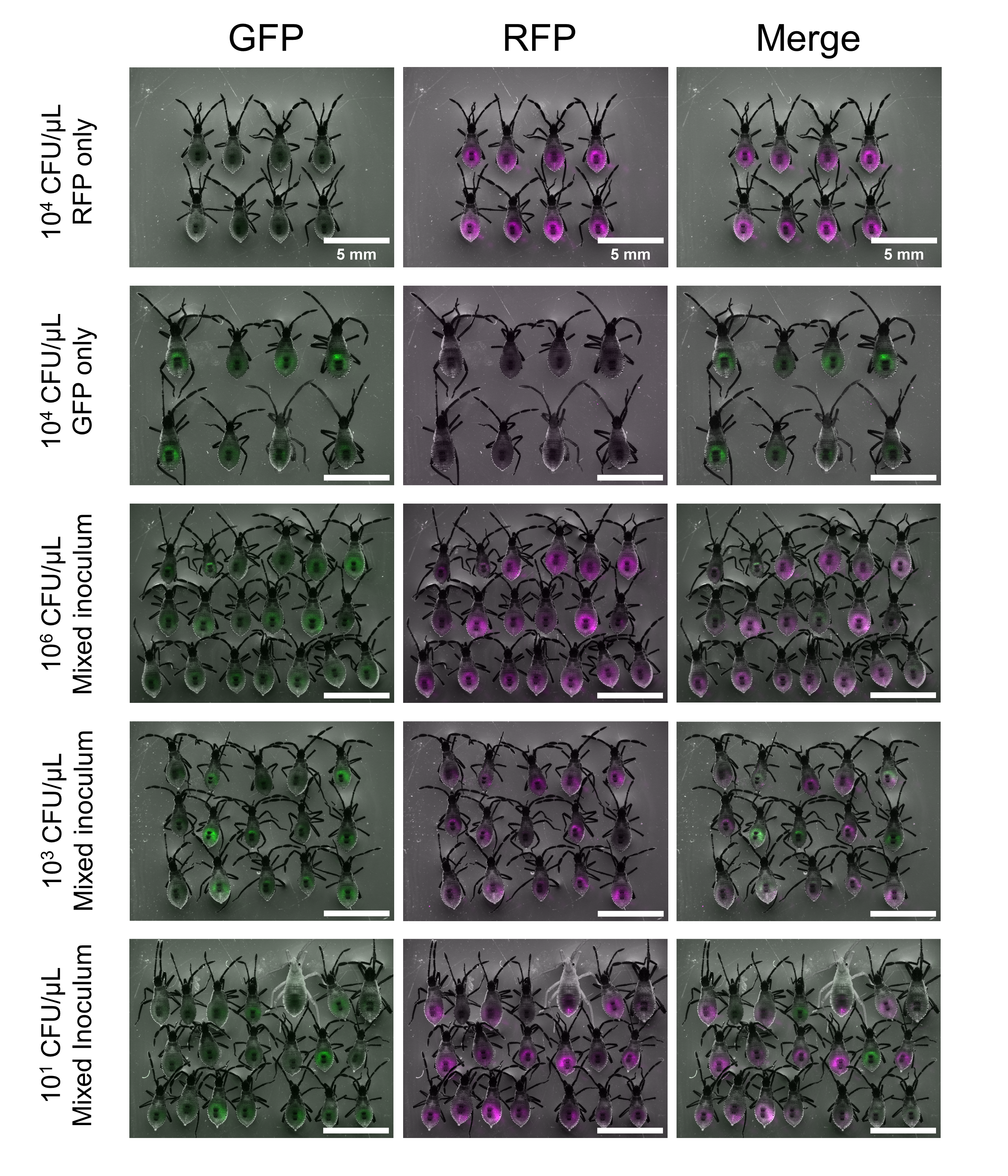

Supplement: S2 Fig — Fluorescence images of nymphs from a single cohort colonized with different densities of GA-OX1 sfGFP and GA-OX1 RFP, ranging from 101 to 106 CFU/μl. Nymphs inoculated with only GA-OX1 sfGFP or only GA-OX1 RFP serve as controls (top 2 rows); the bottom 3 rows show nymphs from mixed inoculation trials (GA-OX1 GFP + RFP). Note that the red fluorescent protein dTomato is brighter in whole-body preparations of nymphs than the green fluorescent protein sfGFP, due to increased absorbance of green light by living tissue [113] and the high stability of free dTomato under physiological conditions. (TIF) [file pbio.3002304.s002.tif]

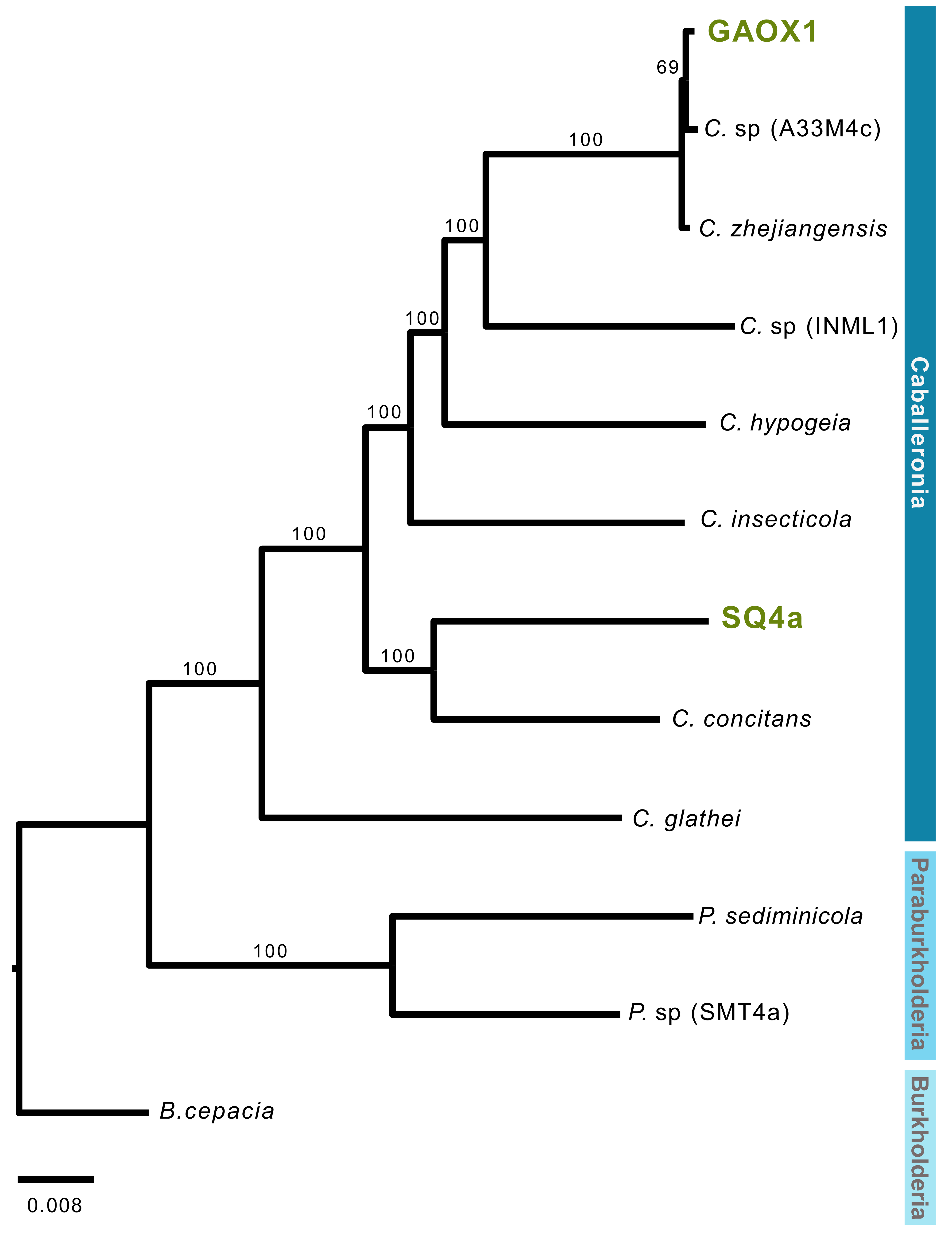

Supplement: S3 Fig — A whole genome-based phylogeny of selected species and previously isolated Anasa tristis symbionts, representing major clades within the genus Caballeronia as defined by Peeters and colleagues [27], including the experimental strains C. sp. nr. concitans SQ4a and C. zhejiangensis GA-OX1. The phylogeny was constructed using RealPhy [114], with Burkholderia cepacia as the reference genome, using default settings except for a gap threshold of 0.1 and setting the model of evolution to GTR. Support values are bootstrap values based on 100 replicates. In addition to SQ4a and GA-OX1, Caballeronia strains A33M4c and IN-ML1 were also previously isolated from A. tristis [7,28]. SMT4a is a Paraburkholderia terricola soil isolate that can colonize A. tristis [28,41]. GenBank assemblies are as follows: GCF_023631065.1 (GA-OX1), GCF_022879815.1 (A33M4c), GCF_022627895.1 (C. zhejiangensis), GCF_023631085.1 (INML1), GCF_001544875.2 (C. hypogeia), GCF_000402035.1 (C. insecticola), GCF_023170545.1 (SQ4a), GCF_001544615.1 (C. concitans), GCF_902833485.1 (C. glathei), GCF_902859805.1 (P. sediminicola), GCF_022879555.1 (SMT4a), and GCA_009586235.1 (B. cepacia). Bootstrapping and tree files are available at https://doi.org/10.15139/S3/YZPBGY. (TIF) [file pbio.3002304.s003.tif]

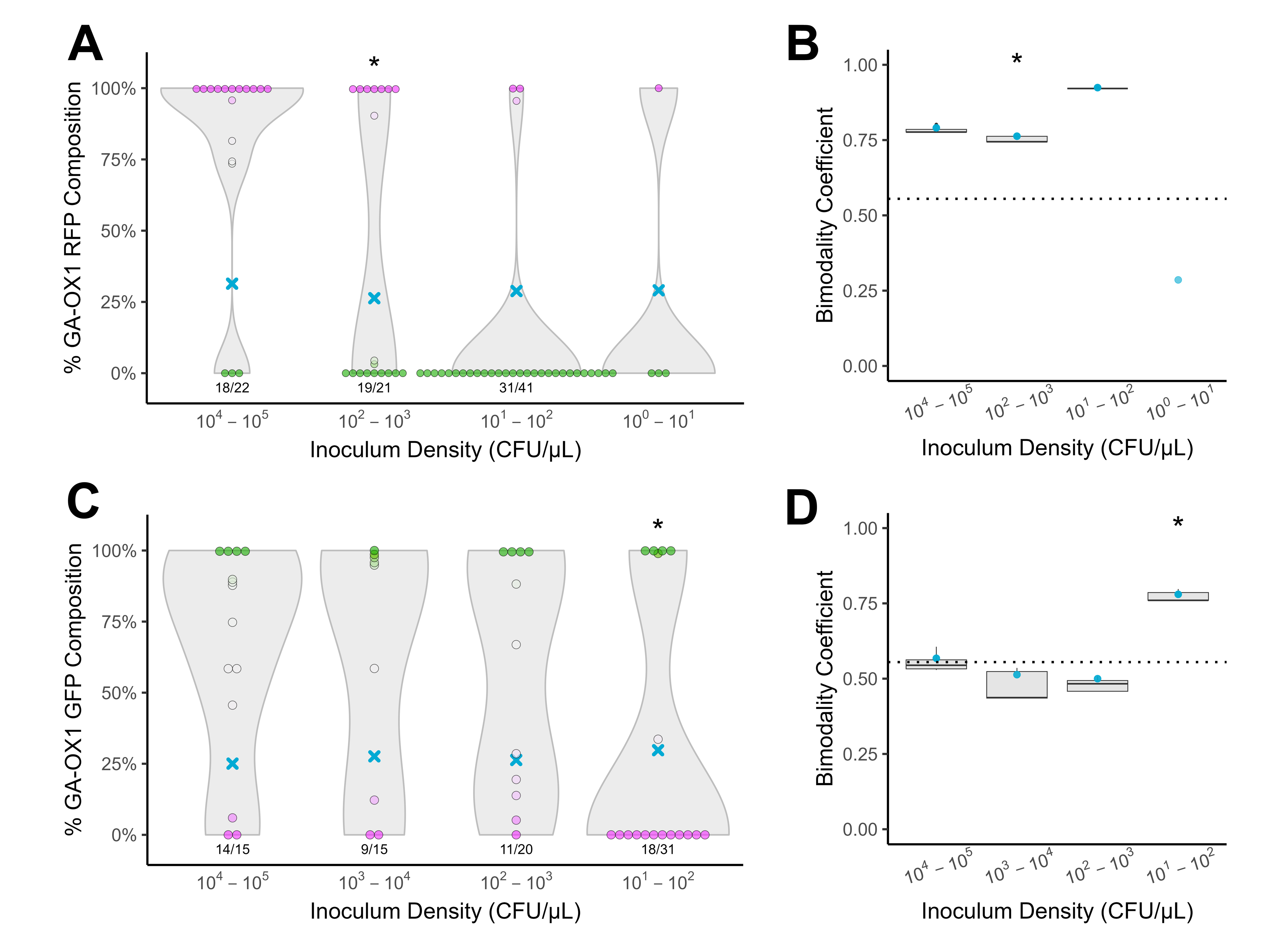

Supplement: S4 Fig — (A) Variable colonization outcomes associated with different transmission bottleneck sizes in two-species co-inoculation, using C. sp. nr. concitans SQ4a sfGFP and C. zhejiangensis GA-OX1 RFP. Blue X marks indicate the mean percent GA-OX1 RFP associated with each inoculum treatment, ranging from 100 to 105 CFU/μl. Points represent individual nymphs, and the color of each point and its position along the y-axis represent the percent relative abundance of GA-OX1 RFP colonies among all fluorescent colonies recovered from each nymph. Magenta points represent nymphs from which only GA-OX1 RFP colonies were recovered, green points represent nymphs from which only SQ4a sfGFP colonies were recovered, and faded magenta/green points represent coinfected nymphs. Violin plots associated with each treatment depict the shape of the distribution in relative GA-OX1 RFP abundance. Below each violin plot, the success rate of colonization is indicated, as the number of nymphs that were successfully colonized with Caballeronia out of all nymphs sampled. These values were not recorded for the 100−101 treatment and thus omitted. Asterisks indicate significantly multimodal infection outcomes as determined by Hartigan’s dip test, at a significance level of p < 0.05. The data underlying this figure can be found in S5 Data. (B) Bimodality coefficients calculated from results in panel A. Large blue dots indicate bimodality coefficients calculated from all bugs in each treatment; boxplots indicate coefficients calculated by jackknife resampling in each treatment. Colonization is bimodal (bimodality coefficient > 0.555) across several orders of magnitude of inoculum density. The 0.555 threshold (marked with a dotted line) indicates the bimodality coefficient associated with a uniform distribution. The data underlying this figure can be found in S5 Data. Note that for the 100−101 treatment, the sample size was insufficient for jackknife resampling. (C) Variable colonization outcomes associated w [file pbio.3002304.s004.tif]

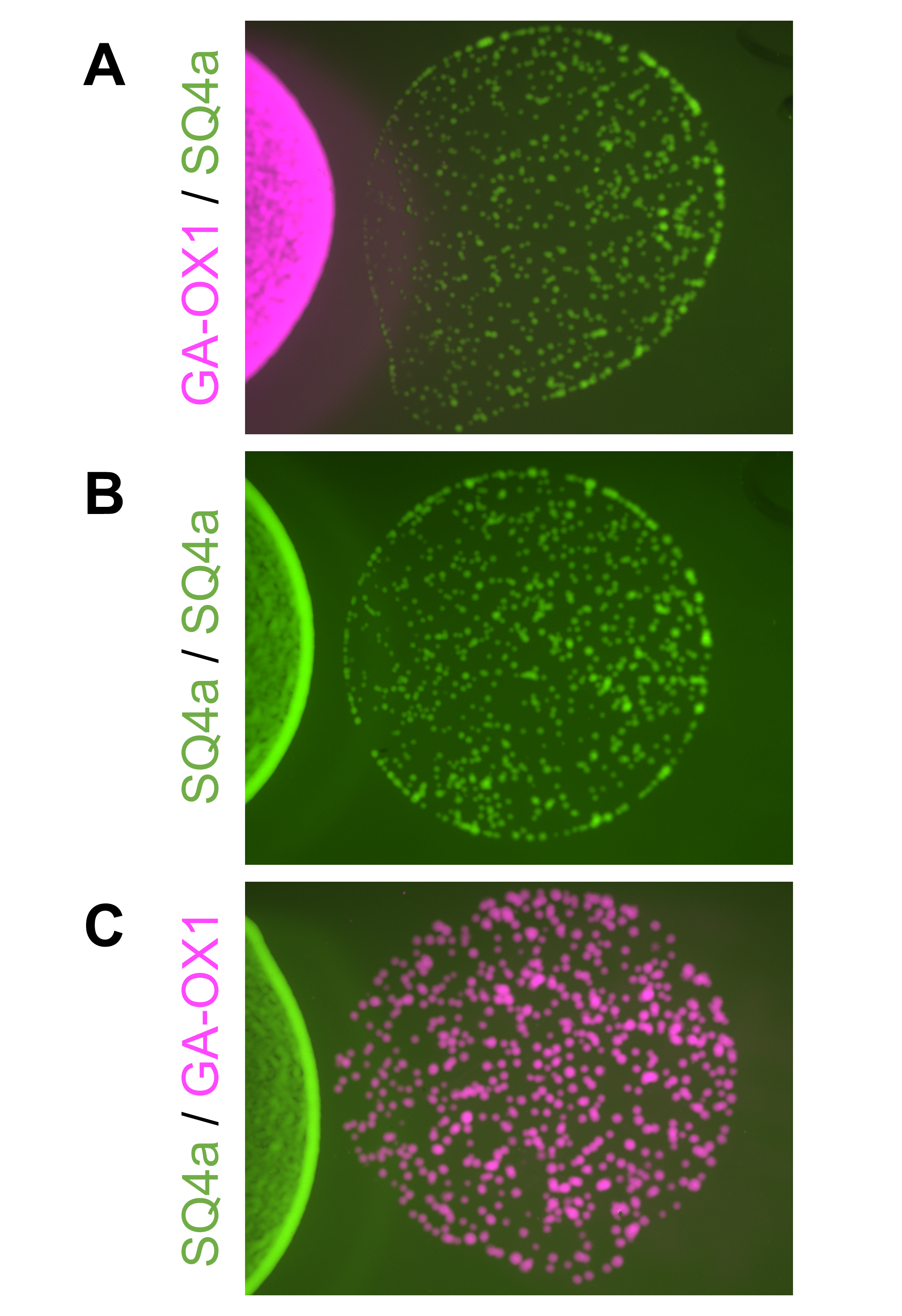

Supplement: S5 Fig — Spots of GA-OX1 RFP and SQ4a sfGFP plated side-by-side at high and low densities on nutrient agar. (A) A dense culture of GA-OX1 RFP spotted adjacent to single SQ4a sfGFP colonies. (B) A dense culture of SQ4a sfGFP spotted adjacent to single SQ4a sfGFP colonies. (C) A dense culture of SQ4a sfGFP spotted adjacent to single GA-OX1 RFP colonies. (TIF) [file pbio.3002304.s005.tif]

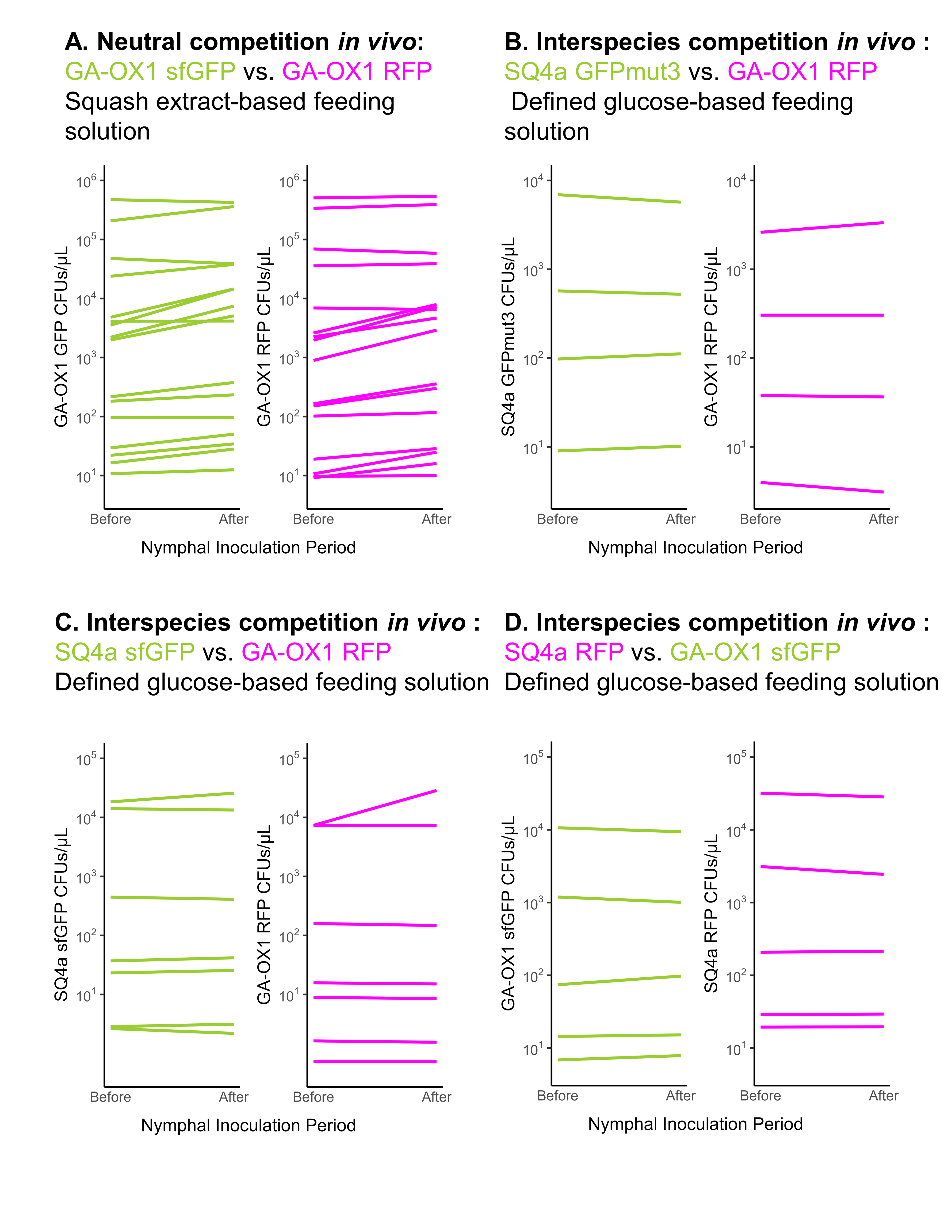

Supplement: S6 Fig — Bacterial strain titers before and after inoculation trials. The data underlying this figure can be found in S2, S4, S5, and S6 Data files. (A) GA-OX1 GFP and RFP (cf Figs 2B, 2C, and S1). (B) SQ4a GFPmut3 and GA-OX1 RFP (cf Fig 3C and 3D). (C) SQ4a sfGFP and GA-OX1 RFP (cf S4A and S4B Fig). (D) GA-OX1 sfGFP and SQ4a RFP(cf S4C and S4D Fig). (TIF) [file pbio.3002304.s006.tif]

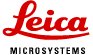

Supplement: S7 Data — Red and green fluorescence intensities measured along digital transects of dissected symbiotic organs (M4s). (ZIP) [file pbio.3002304.s017.zip › S7 Data intensity_data/Dil4-Mix2-10X-withautofocus_Merging_001/LeicaLogo.jpg]

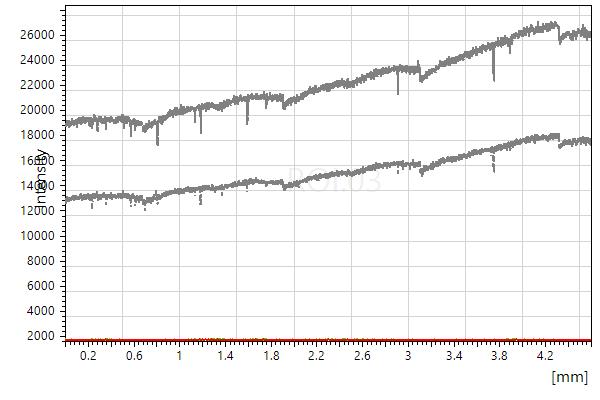

Supplement: S7 Data — Red and green fluorescence intensities measured along digital transects of dissected symbiotic organs (M4s). (ZIP) [file pbio.3002304.s017.zip › S7 Data intensity_data/Dil4-Mix2-10X-withautofocus_Merging_001-background/Chart_2.jpg]

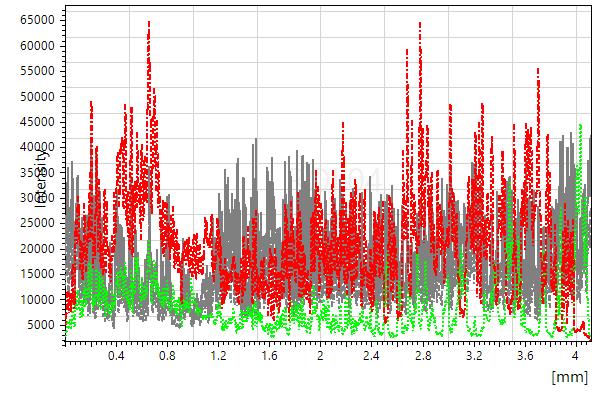

Supplement: S7 Data — Red and green fluorescence intensities measured along digital transects of dissected symbiotic organs (M4s). (ZIP) [file pbio.3002304.s017.zip › S7 Data intensity_data/Dil4-Mix3-10X-withautofocus_Merged/Chart_3.jpg]

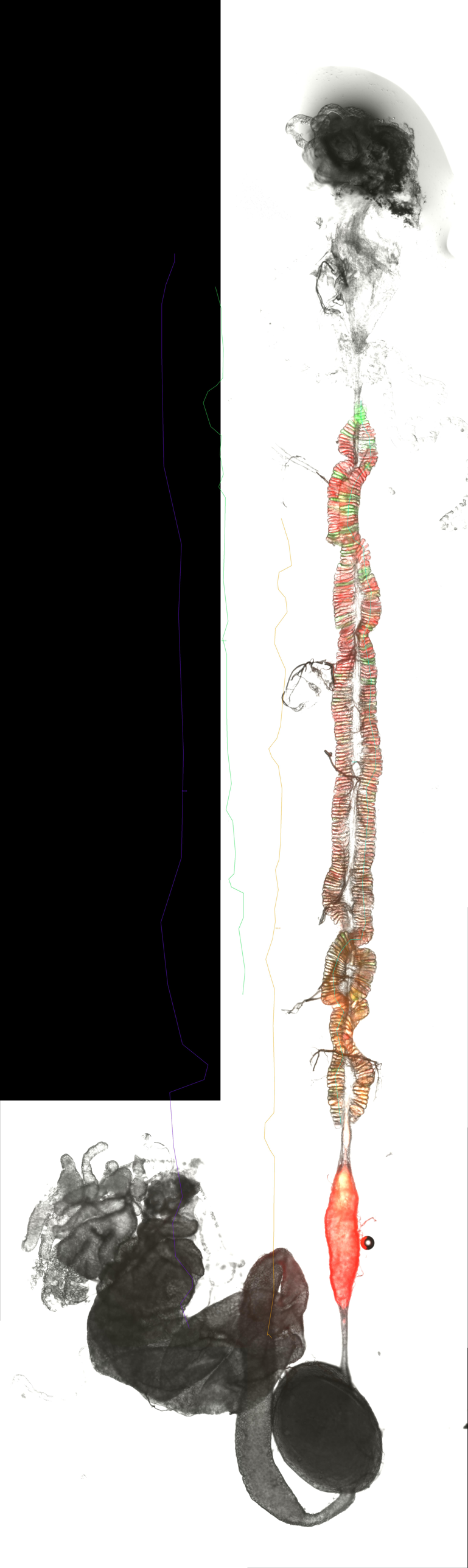

Supplement: S7 Data — Red and green fluorescence intensities measured along digital transects of dissected symbiotic organs (M4s). (ZIP) [file pbio.3002304.s017.zip › S7 Data intensity_data/Dil4-Mix3-10X-withautofocus_Merged/Image1.jpg]

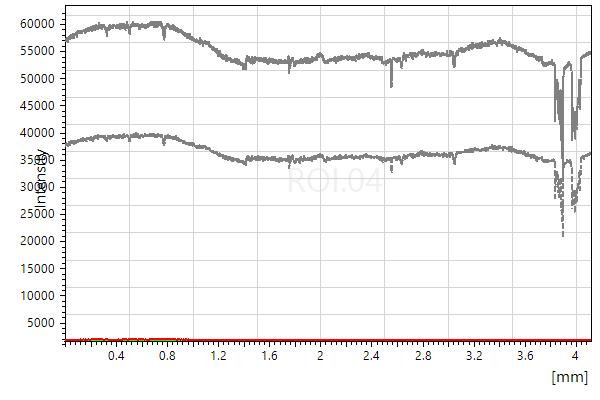

Supplement: S7 Data — Red and green fluorescence intensities measured along digital transects of dissected symbiotic organs (M4s). (ZIP) [file pbio.3002304.s017.zip › S7 Data intensity_data/Dil4-Mix3-10X-withautofocus_Merged-background/Chart_3.jpg]

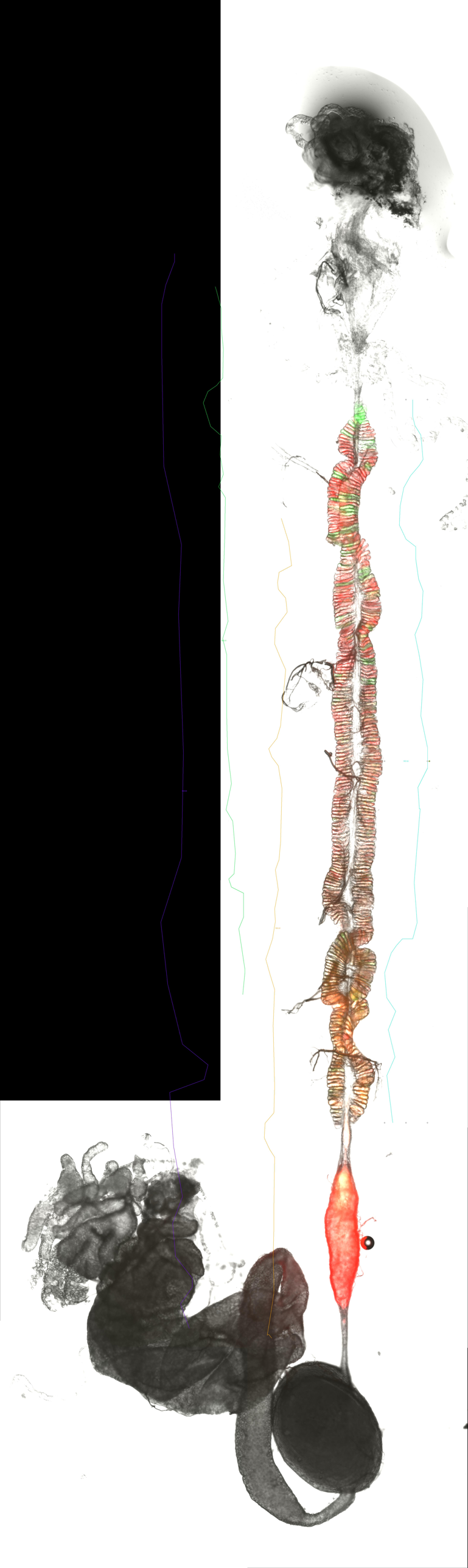

Supplement: S7 Data — Red and green fluorescence intensities measured along digital transects of dissected symbiotic organs (M4s). (ZIP) [file pbio.3002304.s017.zip › S7 Data intensity_data/Dil4-Mix3-10X-withautofocus_Merged-background/Image1.jpg]

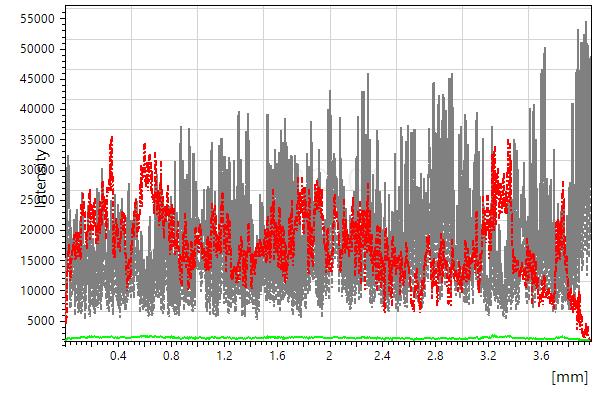

Supplement: S7 Data — Red and green fluorescence intensities measured along digital transects of dissected symbiotic organs (M4s). (ZIP) [file pbio.3002304.s017.zip › S7 Data intensity_data/Dil4-RFPctrl3-10X-withautofocus_Merged/Chart_0.jpg]

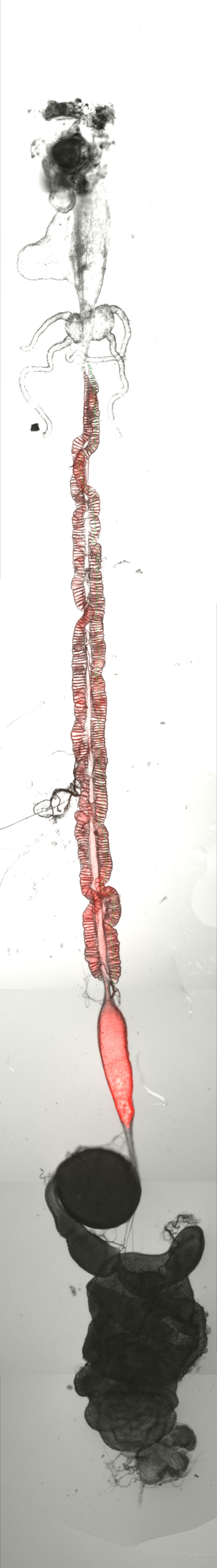

Supplement: S7 Data — Red and green fluorescence intensities measured along digital transects of dissected symbiotic organs (M4s). (ZIP) [file pbio.3002304.s017.zip › S7 Data intensity_data/Dil4-RFPctrl3-10X-withautofocus_Merged/Image1.jpg]

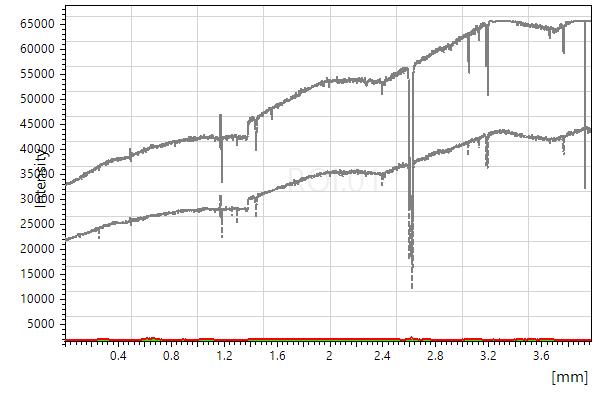

Supplement: S7 Data — Red and green fluorescence intensities measured along digital transects of dissected symbiotic organs (M4s). (ZIP) [file pbio.3002304.s017.zip › S7 Data intensity_data/Dil4-RFPctrl3-10X-withautofocus_Merged-background/Chart_0.jpg]

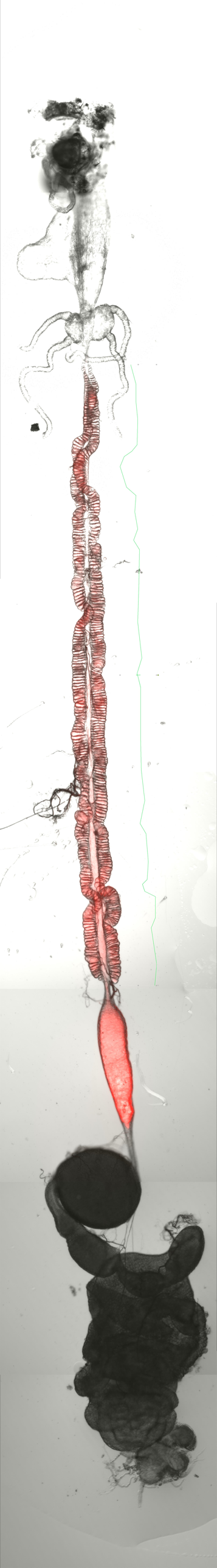

Supplement: S7 Data — Red and green fluorescence intensities measured along digital transects of dissected symbiotic organs (M4s). (ZIP) [file pbio.3002304.s017.zip › S7 Data intensity_data/Dil4-RFPctrl3-10X-withautofocus_Merged-background/Image1.jpg]
