# Supplementary material for: Ecological drift during colonization drives within-host and between-host heterogeneity in an animal-associated symbiont
Source: PLoS Biol. 2024 Apr 25;22(4):e3002304. doi: 10.1371/journal.pbio.3002304 (PMC11075893; doi:10.1371/journal.pbio.3002304)
Supplement: Attachment — Submitted filename: ResponseToReviewers_final.docx [file pbio.3002304.s027.docx]

Reviewer Comments are in **Black** and responses are in **Blue**. Responses are also indicated with “>>.”

Reviewer #1:

Dissecting the causes of variation in microbiome composition across hosts is of broad importance.  With this study, the authors highlight the role of stochastic processes in leading to variation in strain composition both across and within hosts.  With a simple experimental design and beautiful imaging, they show that heterogeneity can be generated across individuals of a host species due to chance differences in colonization of two isogenic strains (other than due to differential markers).  Most interestingly they show that chance processes can overcome a clear difference in non-isogenic strains, if the inoculum density is 1000 cfu/ul or lower.  Additionally, they show that variation can be maintained within an insect host due to differential colonization of the sub-structures (crypts) of the organ that houses the symbionts within the insect gut.

Overall, I found this an interesting and citable study.  My key concern is that the authors do not reconcile their findings of within-host diversity housed in the crypts with the pattern of one strain stochastically dominating in individual hosts.  They must have selectively chosen co-colonized hosts; however, I can't find this explained in the methods or results.

>>We thank the reviewer for their positive comments. In the first part of the manuscript (Figures 2 & 3, Figure S4), data represent random samples from populations of hosts colonized under the conditions indicated; no pre-selection was performed for these samples. In the latter part of the manuscript (Figure 4), to assess spatial patterns of co-colonization, it was necessary to select hosts that were confirmed to be co-colonized. In these data, nymphs were selected for dissection based on a visual screen of co-colonization (both visible GFP and RFP signal being present in the live insect). We now include:

<lines 487-488> “We then intentionally screened individual insects for co-colonization, and only these insects were selected for dissection and microscopy.”

Additionally, there were a few other places in the manuscript where I was unclear about which marker combinations were being presented and how representative the results were.  In Figure 3A, are the in vitro results (n=10) from 1 marker combination or pooled across different ones?

>>In Figure 3A, the *in vitro* results were pooled across different combinations of markers; for co-culture data of two different strains, some co-cultures involved GA-OX1 sfGFP and SQ4a RFP, and others involved GA-OX1 RFP and SQ4a GFP.

In the legend for Figure 3 (lines 198-199), we now accordingly indicate that “Data are pooled across fluorophore reciprocal swaps.”

Further, in the in vivo study, while the bimodal outcome shown in Fig 3C was reproduceable with different marker constructs, the increase in bimodality with the decrease in inoculum dose (Fig 3D) was not, and some of the results seemed strangely skewed (see Figure S4), so it would be nice if this was unpacked a little (even if only in the supplement).

>>We agree that some of our more understated results are the differences between different combinations of marker constructs in the degree of skewness, which was why we felt that these data were worth including in the supplement. We can speculate that there may be small differences between strains, some of which may be attributable to differences between fluorophores and/or lineage-specific tolerances to these fluorophores. Or, this may be due to selection of individual isolates that occurs during preparation of labeled strains. Differences of this nature are very common in experiments that use labeled strains, for various combinations of these reasons. This is by necessity speculative, and as such we do not devote time in the text to dissecting these possibilities.

One of the strengths of this study is that it is motivated by explaining strain diversity observed among hosts in nature.  With that in mind, the manuscript could have benefited from discussing the experimental findings in light of the patterns seen in nature.  For example, how do the inoculum doses given in the experiments compare to those bugs would be exposed to in feces?  Can this be related back to the coinfections observed in 3C?

>>We agree that this is an interesting question, but one that is very difficult to quantify. In previous work, we showed that nymphs feed on feces deposited on environmental surfaces by adults. We have found that freshly deposited, still semi-liquid feces yield anywhere from 1e5 to > 5e6 fluorescently labelled *Caballeronia* CFUs/µL, but it is difficult to determine the volume taken up during feeding, among other factors. Moreover, we also know that bugs can be colonized by presumably small numbers of symbionts from the soil. From these data, the inoculum titers we used in our experiments are within the range of natural variation in transmission bottleneck size. We now indicate this in the text of the Discussion (lines 289-293).

Otherwise, this manuscript was well presented and thoroughly discussed.  It is of value to the literature not so much for its novelty, but because it raises to the forefront and provides a great empirical demonstration of the importance of stochastic processes.  As such, there are some places where the text (and the citation lists) could be streamlined.  Below I flag some places that were confusing or could be improved.

3:  The phrase "providing the raw material for natural selection…." is somewhat distracting for setting the stage and could be deleted

>>This has been removed.

8: better to provide a definition of drift here—saying it "causes stochastics fluctuations" is somewhat confusing

>>This has been replaced with “ecological drift, the noisy fluctuations in community composition due to demographic stochasticity.”

9: add in "we found" after the comma

>>We added “we found that”

21-29:  This paragraph was somewhat awkwardly constructed and could be mostly deleted.

>>Per the reviewer’s suggestion, we have deleted most of this paragraph.

145-146:  Walk the reader through the results a little more.  What would the bimodality coefficient be if the data were normally distributed or if it were skewed to one pole?

>>We now (lines 126-129) give the following explanation to assist with interpretation of the statistic: “The bimodality coefficient is a composite measure of skewness and kurtosis, which is maximized for a distribution with equal weight at the extrema of the data. As this measure can be sensitive to small sample sizes, we also calculated the Hartigan’s dip statistic, which represents a more robust general measure of multi-modality.”

148:  Here do you mean to say that there is "more heterogeneity seen across individual insects"?

>>For clarity, the sentence now reads:

<line 171-173> Fluorescence images of whole nymphs provided qualitative confirmation of our results, with more heterogeneity observed between nymphs at lower inoculum densities (Fig S2).

151-152:  replace "in separate" with "across"?

>>Per the reviewer’s suggestion, the heading now reads:

<lines 175-176> Ecological drift maintains coexistence between competing strains across separate hosts.

166/Figure 3A:  Does the n=10 and what is shown depict two different marker schemes or a specific combination?

>>As explained above and now indicated in the figure legend, the data are pooled across fluorophore swaps.

208:  Tables S2-S4 vs Table S1.  This result could be explained more.

>>For clarity, the text in question now reads:

<lines 232-235> “Bimodality coefficients were consistently higher at high colonization densities in interspecific competition experiments than neutral competition experiments (Figs 3D, S4B, and S4D, Tables S2-S4); this is expected, as neutral competition should produce unimodal populations when colonization rates are high.”

212-214:  clarify that you mean heterogeneity among crypts

>>For clarity, the statement now reads (line 239-240): “…we asked whether symbiont composition might exhibit between-crypt heterogeneity within the host…”

218:  clarify that you mean heterogeneity among crypts

>>For clarity, the statement now reads (line 245-246): “…we would expect greater heterogeneity among crypts within a host when symbionts are subjected to tighter transmission bottlenecks during host colonization.”

255: delete "between hosts" or move to previous line

>>We thank the reviewer for catching the error. The text has been removed.

259: remove citations

>>The citations have been removed.

288-294:  I found this interesting, but it is a fair amount of discussion for something not examined in the manuscript.

>>We appreciate the feedback. Other reviewers have also requested a shorter discussion. To that point, we have streamlined this paragraph while still trying to convey that our system is tractable because of its simplicity and that our results are generalizable to more complex microbiomes despite this simplicity.

337-339:  It's not really clear from what you've written how these results vary to the results found in vitro or why host control would lead to coinfection in anterior crypts.

>>We have revised this paragraph to more clearly communicate the expected relationship between inoculum density and population admixture *in vivo.*

378-387: This information would better go in the legend for Supplemental Figure 3 as the data is not presented in the main manuscript.

>>Per the reviewer’s suggestion, we have moved the paragraph to the caption for Supplemental Figure 3.

391-396:  It's not clear whether this information is needed.  Was the rifampicin resistant mutant in this study?  Shouldn't the GFPmut3 be listed in Table 1?

>>For clarity, we have removed these lines from the paragraph. Some of this information is now contained within Table 1.

434:  Are the 4 combinations the ones shown in Fig S6?  From the wording here, it sounds like you have both species in self vs. self pairings and then variably labeled in the cross species pairings?

>>The four combinations you refer to here deal with growth experiments purely *in vitro* (Figure 3A) in which we do have both species in self vs. self pairings and in cross species pairings in which they are cross labeled. We have clarified in the headings in Figure S6 that the data therein pertain to inocula used in *in vivo* colonization experiments.

435: Clarify that self vs. self was only done with GA-OX1, not with both strains.

>>We have not changed the text as it is correct- both SQ4a and GA-OX1 were grown in self vs. self liquid cultures.

452:  what volume of 1x PBS

>>The volume is now indicated.

457:  Was the blue dye used in both feeding solutions?

>>Yes- this is now indicated.

460: Figure S4 does not appear to be the correct figure here

>>The statement should have referred to Figure S6 – this has been corrected.

523: Where the Fst presented in the main text?  Perhaps you could mention and interpret the Fst values in each section of the results. Or here make a general conclusion about how it relates to the other metrics.

>>The Fst metric proved to be redundant with the other measures of multimodality and is not presented in the main text. We have removed the relevant statements from the Methods.

528:  It's not clear what the 3 p-values refers to.

>>The p-values referred to are for the dip test. We have revised the indicated section for clarity.

898:  Should refer to figure 2B?  Although the number of points don't seem to match what is shown in 2B?

>>Figure 2B depicts the same data as Figure S1B, but with all trials binned into discrete treatments for analysis and illustration. In Figure S1B there is substantial overlap between the points at the extrema of 0% and 100%, due to the lack of jittering. Points were not jittered to avoid confusion between data from separate trials with similar inoculation treatments. This is now indicated in the figure legend.

916-917:  These lines should be deleted and replaced by text that helps to explain the bottom 3 rows of the figure.

>> We have removed irrelevant text and clarified the contents of each row.

920:  Provide more information here on how the phylogeny was constructed.

>>We have added details: “Whole genome-based maximum likelihood phylogeny was constructed using RealPhy, with *Burkholderia cepacia* as the reference genome, using default settings except for a gap threshold of 0.1 and setting the model of evolution to GTR. Support values are bootstrap values based on 100 replicates.”

Reviewer #2:

In this study, the authors describe an elegant set of experiments examining the role of ecological drift in the colonization of the M4 organ of the squash bug Anasa tristis by Caballeronia symbionts. Using controlled experiments with fluorescently labeled Caballeronia strains, they find that variation in community composition between hosts can arise as a result of stochastic colonization dynamics and influenced by inoculum size. Interestingly, they also find that the strength of this effect varies spatially along the length of the digestive tract within a host. Overall, I think this study is interesting and may be worthy of publication in PLOS Biology, but some additional detail and context on the authors' results, along with some adjustments to the framing of the manuscript, would greatly strengthen the publication. 

Major comments: 

1. Some of the authors' claims are over-stated and over-generalized. For example: 
    -In several places it is implied that the authors results' are generalizable beyond the squash bug system. For example in lines 51-53: "To illustrate the importance of ecological drift during the establishment of even highly specific symbioses, we employ the squash bug, Anasa tristis (Fig 1A), as a model." In fact, the delayed colonization of the organism and the generalist ability of the Caballeronia to live in both host-associated and free-living environments could both be important factors influencing the size of the effect observed by the authors. I recommend revising the text to limit over-generalizing and in particular editing the title to include the name of the study species.
   -Relatedly, please clarify in the abstract that the focus is on close host-microbe symbioses rather than all host-microbiome interactions.

>>It has been repeatedly established that drift and other stochastic processes are important for ecological dynamics of multi-species systems, including but not limited to host-microbe communities. The novelty of this paper is specifically in demonstrating application of this ecological rule to a specialized symbiosis, a scenario in which host control might be expected to over-ride signatures of stochastic processes. This is relevant to many other natural systems, including models of symbiosis, such as the squid-*Aliivibrio* and legume nodule symbioses. These are also systems in which within and between-site diversity of the symbiont might be affected by stochastic processes during colonization. We have provided Figures 1D and 1E as conceptual figures, on the recommendation of Reviewer 3.

>>We have clarified the focus of this manuscript in the Abstract.

>>We feel that the current title adequately represents the contents of the manuscript.

    -Lines 12-14 of the abstract: How do the authors' results show that ecological drift is responsible for the within-host heterogeneity, as opposed to other possible host contributors? 

>>Our experimental design intentionally rules out a number of host contributors, including host lineage-specific effects, batch effects, and differences in host benefit from different symbiont strains. This design mimics classical designs used to study analogous processes in host-parasite dynamics.

2. At the same time, for the authors' main results on colonization outcomes in individual insects, it is not very clear what an alternative outcome would look like - increasing bimodality seems basically inevitable with shrinking input population sizes. It would be helpful if the authors could provide additional context on what specific alternative hypotheses could be considered, and how the expected results of their experiments might differ under an alternative hypothesis.

>>We now highlight two additional hypotheses and their implications early in the Results (lines 116-119): “By contrast, when host control determines colonization, altering the inoculum size should have minimal impact on community composition across hosts. Additionally, if strong competition between symbionts determines the outcomes of colonization, individual hosts should be mono-colonized across a broad range of inoculum densities.”

3. Please make sure all terms are clearly and specifically defined in the introduction. For example, the authors seem to use "composition" to refer to microbial taxonomic composition, or which species/strain is dominant (as opposed to other ways we could describe the composition of a microbial community). I would probably also define "heterogeneity" as the authors are using it here.

>>Where relevant, we have defined these terms to the best of our ability.

4. The bimodality coefficient is a nice statistic to quantify the stochasticity the authors are looking for, but it's not clear how robust this metric is to differences in sample size and individual outliers. I recommend the authors perform a bootstrapping or subsampling analysis on their data in order generate confidence intervals/error bars for each of their bimodality coefficient estimates.

>>We have carried out the suggested analysis, using a jackknife approach, which is more relevant here given sample sizes of these experiments. Results are now presented in the text.

5. Figure 1C needs to have a taxonomy legend. As is, it is not possible to determine what taxa are shown and whether it supports the authors' point.

>>As indicated in the legend, all reads in these data belong to *Caballeronia* species – the specific species identifiers do not add information. The point of this panel is to show the degree of co-colonization by very closely related members of the same genus within the crypts of wild individuals.

6. There are some inconsistent or puzzling results reported that are not addressed in the text. Specifically, what do the authors make of the variability in the fraction of colonized insects, and the sometimes sizable share of animals that are not colonized in any single experiment?

>>By using the natural route of inoculation (e.g. feeding), some individuals will not consume sufficient inoculum and will not be colonized. The same effect is observed when colonizing nymphs from fecal samples, a known natural route of inoculation.

Secondly, it appears that increasing bimodality with inoculum dose was not observed in Figure S4A-B. What might explain this discrepancy?

>>The drop-off in bimodality at the lowest inoculum in panels A-B is due to the low sample size; the coefficient used is known to be sensitive to this factor.

7. I have several follow-up questions about factors that might influence the observed phenomena. I don't believe the authors necessarily need to experimentally test all of these factors prior to publication, but if they have explored or considered the effects of any of these, it would be useful to include and/or mention in the discussion. 
    -Are these bacteria motile? Could there be transmission between crypts? Does the duration of the experiment influence the final outcome?

>>These bacteria are known to be motile in culture, but it not clear how much if any movement there is between crypts *in vivo* after the initial inoculation. The possible effects of transmission between crypts are explored in the Discussion.

    -Are there differences in the total number of colonized bacteria between hosts and inoculum doses? A consistent maximum population size across the individuals with different "winning" strains would provide additional support for the importance of ecological drift.

>>Because the hosts were allowed to naturally feed, we do not know precisely how many bacteria each host consumed.

    -Relatedly, the data on spatial heterogeneity within-host would be easier to interpret if it were presented in comparison with the spatial distribution upon colonization with a single labeled strain alone. Could it reflect a gradient in the bacterial carrying capacity from anterior to posterior due to nutrient availability or some other environmental factor?

>>Reviewer 3 asked a similar question about more clearly demonstrating the spatial distribution of both fluorophores within the host. We have improved figure 4A by depicting single and merged fluorescent channels without differential interference contrast. Moreover, in figure 4C, we depict a ratio in RFP and GFP intensity, which is not sensitive to any changes in carrying capacity along the anterior-posterior axis.

    -For the in vitro competition experiments, is there any effect of either a) spatial heterogeneity (such as not fully blending or filtering the zucchini) or b) culture volume on competition outcomes?

>>We did not assess spatial heterogeneity *in vitro*, which took place in shaking squash extract cultures without spatial heterogeneity. The culture volume was chosen for compatibility with the multi-well format used; we do not know whether changing the volume substantially would alter these results.

8. Please make sure to make data and code fully available as promised.

>>We will make data and code available in line with *PLOS Biology* guidelines.

Minor comments: 

1. Lines 253-255 is confusing: "Previous research on a suite of closely related insect-Caballeronia symbioses has uncovered both heterogeneity in symbiont composition and low diversity of symbiont populations within hosts between hosts" - is it within hosts or between hosts?

>>This should be “within hosts” – the error has been corrected.

2. The gray "x"s in the violin plots are very hard to see in some plots - it would be helpful to make them a different color not currently used in the visualization (maybe red or blue).

>>We have made the “x”s blue for clarity.

3. Figure 4C is a very nice result but would benefit from being larger and higher resolution.

>>We will provide images at the size and resolution required by PLOS Biology guidelines.

Reviewer #3:

It has been recently recognized that there are many plant-feeding heteropteran insects which establish gut symbioses through environmental transmission with very high specificity in acquiring a certain group of bacterial species (one or few at a time), in contrast to more complex compositions and many lineages of bacteria co-existing together in many other insect gut systems. Either way, how do these assemblies of bacterial associations form and what determines the dynamics at the onset have been difficult to study in many insects. 

In this study, Chen, J. and the colleagues reached some empirical evidence of symbiont heterogeneity formation between and within host individuals using the squash bug Anasa tristis and its gut symbionts Caballeronia, an emerging system of host-microbe associations. Detailed comparisons and observations of establishing symbioses on closely related yet functionally equivalent strains/species of microbes have been rarely tested in other insect-microbe studies, thus novel and interesting as the authors implied. The authors revealed the competitions between strains of the slightest variation naturally led to more doubly-infected insects, but also ecological drift can produce bimodality under the very tight bottleneck, which is somewhat predictable. However, interestingly, that drift (with unforeseen bacterial behaviors) in colonization can be more pronounced by competitive strains of two species of beneficial symbionts, leading to more singly-infected insects even at higher
densities. In addition, by dissecting and observing the colonization of insects the authors found within-individual colonization heterogeneity among different parts of crypts, which might explain the heterogenous compositions of gut symbionts from field-collected insects in other Caballeronia-stinkbug symbioses. 

The authors conducted very well-designed experiments, namely, preparation of fluorescently labelled isogenic strains of two bacterial species, inoculations of symbionts and quantification by CFU counts of labeled colonies, a novel observational approach using whole insects and fluorescent bacteria, and several in vitro and in vivo competitive assays taking advantage of the above system. With statistically robust and sufficient analyses, the authors collectively unraveled appearance of bimodality of one-on-one symbiont competitions under bottlenecks also supported by supplemental data, presenting us insightful picture of the phenomena that can be also tested by the others. This study would illuminate the importance of analyzing symbiont transmission dynamics of gut microbes not only from rather mechanism-oriented insect symbiosis field but can be highlighted also from another perspective especially involving theoretical models of species co-existence, as the authors claim. 

Most of the results, methodologies, and figures were easy to follow and very informative. All the experimental systems have been well established previously by the authors, hence no concerns.

Overall, in my opinion, the manuscript is bit too long especially in the discussion trying to cover excessive references, not explaining much in the text in contrast to very simple results.

>>The revised version of the manuscript has been shortened and re-worked for clarity.

Why not make a conceptual model figure at first?

>>Figure 1 now includes a conceptual model for clarity.

The discussion should be revised after illustrating the basic concept/predictions in the introduction, because it was not straightforward how the authors integrated all these ideas such as population bottlenecks, neutral theory of biodiversity and observed heterogeneity in this seemingly a very empirical paper. Also, I raised more specific issues and modifications to be made in the minor comment section as follows, even though I would expect they require a few more rounds of experiments and observations.

>>We have revised the Discussion for clarity.

This manuscript demonstrated a degree of heterogeneity formation in symbiont community assembly employing microbe-microbe-host interactions; however, its impact is still somewhat limited considering its highly specific symbiosis and studying only initial stages of insect life cycle for discussing their evolution or selection(s) on the whole system of host-microbe interactions. This study will likely appeal to the other scientists in the same field, only if revised well, also potentially stimulating more research on such interactions in the context of microbe-microbe competitions involving selections by and on the animal/plant hosts.

>>While we use a specific symbiosis in this paper, the same processes that are mechanistically relevant in this system apply to a range of other symbioses involving both animal and plant hosts, including the canonical squid-Vibrio and legume nodule symbioses. We therefore think that the results presented here are broadly applicable beyond the insect model.

More specific comments on the manuscript

L7 "…strain diversity…"
It is far from "diversity" but rather strain variation within a species.

>>We now refer to this as “inoculum composition”.

L11 "unrelated strains"
Should be <unrelated strains of two distinct species>.

>>We now refer to this simply as “competing strains”.

L21 "…play host to…"
I would avoid using this phrase in this context.

>>The line in question has been removed in the revised manuscript.

L71, Fig.1C "…within 9 bugs…"
Insert adult before "bugs".

>>We have made the suggested change.

L103-L104 
I do not see any remarks explaining the bottlenecks and resulting bimodality of the population structure at least the neutral model discussed in this paper. Please further clarify or refer to another specific citation.

>>The initial citation was incorrect and has been changed to refer to the correct work by this author.

Fig.2A 
Why are some bacterial cells depicted by two distinct colors lighter compared to the others? Do they mean different expression variants of the same fluorescent proteins within the strain, not clearly determined by genomic sequences?

>>This was intended to produce an effect of depth in the test tubes. We have made all cells of the same type the same color for clarity.

Fig.3B and 3C 
The figure order seems to be reversed in the text.

>>We have corrected the ordering in the text.

L202-206
One should avoid using "superior" or "weaker" mentioning each competitor inferred from the in vitro experiment for this line yet, because it is misleading when these strains are facing here a completely different setup (host environment) of in vivo experiment. It only can be speculated that the GA-OX1 may have a capacity to eliminate SQ4a at high density in vitro or before M4, likely producing some toxic substance to SQ4a under the regulation of quorum sensing or something. Following the previous reports, highly dense populations of environmental bacteria are not really required to establish symbioses by the stinkbug hosts, so these results also suggest that both strains have no superiority in the competition of initial host colonization in more realistic circumstances (lower density) in comparison with apparent artificial condition (extremely high density). The authors need to conduct colonization assays mixing lower density GA-OX1 and higher density SQ4a or vice versa, if they want to discuss the "superiority" of GA-OX1 strains.

>>We have removed the descriptors for clarity.

L224-226 "However, there is …(Fig.4B)"
The figure panels do not really convey information that the authors are trying to show. Revise all of them, for example separating GFP and RFP images and add as a supplemental figure?

>>The data in Figure 4C show quantification of the RFP and GFP channels, and these data clearly show the anterior-posterior gradient described. For illustration, we have modified Figure 4A to depict one example specimen with channels separated.
 
L246-250, Fig4C
The colors of these lines have not been explained. Please clarify.

>>We note in the figure caption that “different colored lines represent the trajectories of these values [that is, the ratio of normalized RFP intensity relative to normalized GFP intensity (left) and variance in this ratio within a sliding window (right)] associated with each nymph.”

L305-318
I agree with the authors that the heterogeneity is generated within the squash bug hosts temporary, however, it is not clear yet how these spatial structures are to be maintained and each singly- or doubly-colonized crypt persists along with their host life cycle especially at later stages, when the previous reports have observed more biased colonization densities in field-collected adults (Itoh et al. 2014 AEM; Kuechler et l. 2016 ME; Ohbayashi et al. 2022; this study's Fig. 1C) and host molecular/digestive mechanisms that potentially regulate the whole cell population (Ohbayashi et al. 2019 ISME J) in addition to between-strain competition. This study only surveyed very early nymphal stages of the insect after inoculation of only two strains at an equal ratio, so we are not certain how this stochastically produced heterogeneity and compartmentalization persist within host bugs, and if they are significant biologically. 
Analyze these heterogeneity in later instars/adult insects, or tone down?

>>Figure 1C shows a high degree of co-colonization in wild adult individuals, with a wide range of variation. This is the pattern we are attempting to explain as, at least in part, the product of stochastic processes. The bias can be produced by niche-based or other competitive processes, both inside and outside the host; this is in no way inconsistent with the hypothesis that stochasticity can contribute meaningfully to distributions of diversity between hosts. The indicated text refers to establishment of the observed pattern, which is supported by the data shown.

L319-329 
Before expanding to other biological systems or evolutionary significance of symbiont heterogeneity, the authors should clarify the fitness effects of such heterogeneity on host insects, the ultimate selection acting on the host-microbe associations, in their own system as is discussed later in the next paragraph.

>>The paragraph in question has been removed.

L348-350 "We post that … these same processes"
I am not convinced. Here, the ecological drift in this study only acted on initial stages at and after insect colonization, and the authors also chose the well-preforming successful symbiont strains for the insects. So, strictly speaking, "selection" (if I understood correctly) has not been overcome.

>>We agree that the language used was too strong, as selection in this host-microbe symbiosis is undoubtedly at play, and that the use of “successful” symbionts limits our ability to discuss the effects of host selection. However, selection is weakened by drift, as with any mechanistic property of a system that limits interactions between potentially competing strains. The language in this section has been revised accordingly.

We can also expect the hosts have evolved many ways to nurture, control, terminate and then refresh the specific beneficial symbioses at later stages in their life cycle, such as sanctioning, seen in other systems, sort of social cooperation and polishing discussed by the authors above L321-325 but would be performed by hosts here.

>>We agree that this may be the case but is beyond the scope of the present study.
